# Chamaejasmenin B, an Inhibitor for Metastatic Outgrowth, Reversed M2-Dominant Macrophage Polarization in Breast Tumor Microenvironment

**DOI:** 10.3389/fimmu.2021.774230

**Published:** 2021-12-28

**Authors:** Qi Li, Lidong Sun, Li Liu, Qingsen Ran, Xinke Du, Qing Yang, Yajie Wang, Yujie Li, Ying Chen, Xiaogang Weng, Weiyan Cai, Xiaoxin Zhu

**Affiliations:** Institute of Chinese Materia Medica, China Academy of Chinese Medical Sciences, Beijing, China

**Keywords:** Chamaejasmin B, breast cancer, macrophage polarization, metastatic outgrowth, mTOR

## Abstract

Metastasis is a multistep process that depends on the interactions between tumor cells and their microenvironment. Macrophages in the tumor microenvironment show high polarization plasticity and have a paradoxical role in cancer progression. Hijacked by tumor-promoting signals, the polarization status of macrophages was pathologically disturbed and believed to be the decisive mechanism forcing the progression of metastasis. In this study, we explored the immunological activity of Chamaejasmin B (ICJ), a previously proved inhibitor for metastasis, in macrophages from metastatic microenvironment. When intravenously injected of 4T1 cells in mice, ICJ significantly inhibited its metastatic outgrowth. Taking tumor cell and macrophage as a functional integrity, an adoptive transfer model was established *in vitro* to exclude the direct effect of ICJ on tumor. The findings suggest a dual influence of ICJ on both tumors and macrophages, as indicated by the rebalance of macrophage polarization and suppression of clonogenic potential in tumor cells. Mechanistically, ICJ redirected M2-dominant polarization of tumor-associated macrophage in an IL-4-mTOR-dependent manner. Collectively, our study revealed that ICJ rebalanced macrophage polarization in malignant microenvironment and showed promising effect in suppressing metastatic outgrowth in breast cancer model.

## Introduction

Metastasis is the key event in malignancy and is responsible for most cancer-related deaths. It consists of multistage steps that involves the invasive growth of the primary tumor, circulatory dissemination, and secondary colonization (1, 2), among them, metastatic outgrowth, supported by the tumor microenvironment (TME), contributes greatly to relapse and poor prognosis of patients (3, 4). Notably, due to early-phase dissemination and long-time dormancy, current treatments will be ineffective once tumor cells have colonized into foreign organs (5–7). Therefore, effective drugs targeting metastatic outgrowth are urgently needed, especially for the late-phase intervention of metastasis.

Increasing evidence suggests that the TME, in which the immune properties are pathologically disturbed, contributes greatly to malignant progression (8, 9). Tumor-associated macrophage (TAM), which is the most abundant immune cell in the TME, has both anti- and protumor effects (10) and is negatively correlated with clinical outcomes (11). Physiologically, macrophages are pruned to be in a classic activation status (M1 polarized program), characterized by the elevated proinflammatory activity, the enhanced antigen presentation, and tumor-lytic potential (12, 13). In contrast, during tumor progression, TAMs resemble alternatively activated M2-like macrophages that contribute to malignant angiogenesis, tumor cell intra/extravasation, and outgrowth (14, 15). In both the spontaneous genetic model of breast cancer metastasis and the orthotopic syngeneic transplant model, breast cancer progression and metastasis can be hindered *via* the modulation of TAM polarization (16). Therefore, macrophage polarization is an appealing target for metastatic intervention and is a common focus of both experimental and clinical studies (17).

The mammalian target of rapamycin (mTOR), which affects cancer progression and TAM polarization, contributes to the regulation of metastasis in many cancers (18, 19). The pathological activation of mTOR causes M2 over-polarization, which promotes metastasis in breast cancer cells (20, 21).

Chamaejasmin B (ICJ) is a natural compound extracted from *Stellera chamaejasme* L. (SCL), an herb that is historically thought to inhibit tumors. Our previous studies have suggested that ICJ is a nontoxic and potent antimetastatic agent for breast cancer treatment (22, 23). However, the exact mechanism by which ICJ inhibits tumors and its interactions with the TME remains unknown. In our study, we demonstrated the immunological effects of ICJ on TME-mediated macrophage polarization. More importantly, by targeting metastatic outgrowth, we revealed a novel mechanism through which ICJ can be used in late-phase interventions for metastasis. This new mechanism widens the time window for early-phase metastatic intervention.

## Materials and Methods

### Preparation of Drug

Lyophilized powder ICJ was extracted and purified by DaLian Institute of Chemical Physics, Chinese Academy of Science. The powder was dissolved with normal saline containing 10% DMSO and 1% Tween-80.

### Cell Culture

RAW 264.7 and MDA-MB-231 cell were purchased from the American Typical Collection Center (Gaithersburg, MD, USA), 4T1 cells with stable firefly luciferase expression were provided by Caliper Life Sciences (Hopkinton, MA, UA), and were cultured in RPMI-1640 (C11875500bt, Gibco, Waltham, MA, USA) or Dulbecco’s modified Eagle’s medium containing 10% fetal bovine serum (SH30070, Hyclone, Logan, UT, USA), at 37°C incubator with 5% CO_2_.

### MTT Assay

In MTT assay, the cells were paved onto 96-well plates at 3 × 10^3^ cells/well. After incubation for 24 h, the drugs at serial concentrations were added into each well. At the indicated time points, 100 μL (3-(4,5-dimethylthiazol-2-yl)-2,5-diphenyltetrazolium bromide (MTT, Sigma, St. Louis, MO, USA) was added and reacted for 4 h and following dissolution in DMSO. The optical density value in 570 nm was detected on a microplate reader.

### Flow Cytometry Assay

We established a transferred conditioned medium model *in vitro* to analyze the effects of ICJ on macrophages under tumor-associated conditions using flow cytometry. Cells were detached and stained for surface antigens with FITC-conjugated antibody (F4/80^+^, Proteintec, Rosemont, IL, USA), CD206^+^ (Proteintec, USA), and PDL1 (Proteintec, USA). The cells were then incubated in the dark for 30 min at 4°C. Finally, the cells were analyzed by flow cytometry.

### Western Blot Assay

Cells were routinely digested and seeded onto six-well plates at a density of 2 × 10^5^ cells/well. Samples were harvested at the indicated time points followed by the addition of a lysis buffer. The protein concentration was quantified by BCA (Thermo Scientific, Waltham, MA, USA). The same amount of protein was separated by SDS-PAGE and then transferred to a polyvinylidene fluoride (PVDF) membrane (Millipore, Burlington, MA, USA). After blocking with 5% BSA solution, the PVDF membrane was incubated overnight at 4°C with the following primary antibodies: p-AKT, p-mTOR, p-S6k, TSC1, p-TSC2, iNOS, and ARG1 (purchased from Cell signaling Technology, Danvers, MA, USA, Abcam, Cambridge, UK, and Sigma, USA, respectively). The membrane was then washed three times with TBST and incubated with secondary antibody-linked HRP. Densitometric quantification was carried out using chemiluminescence detection reagents with β-actin (Sigma, USA) as a loading control for whole-cell lysate experiments. The gray values were analyzed by ImageJ software, and all images were normalized.

### Colony Forming Assays

Cells were digested and seeded onto six-well plates at a density of 2 × 10^5^ cells/well. Drugs were added after 24 h, and the cells were cultured for 15 days. The clones were fixed and treatment with crystal violet. The amounts of clones were then quantified (independent clone diameter ≥0.5 mm).

### Animal Assays

The mice were randomly grouped, and the mouse model of pulmonary metastasis was established through vein tail injection. Metastatic 4T1 cells with stable firefly luciferase expression were provided by Caliper Life Sciences (Waltham, MA, USA). The cells were resuspended in PBS and injected into 8-week-old female BALB/c mice at 1 × 10^4^ cells/mice (This design from tail-vein injection model has been optimized based on our small-animal imaging platform for the best monitoring quantity). During drug administration, the metastatic progression was dynamically visualized and quantified using an IVIS Spectrum Imaging System (Caliper Life Sciences) until the model group died of metastasis. Before being sacrificed, the mice were injected with 200 μL D-firefly potassium salt (reconstituted in sterile PBS to a concentration of 15 mg/mL). The mice were then anesthetized by isoflurane and imaged using a small-animal imaging system.

### Histological Analysis

The lung tissues of the mice were collected and fixed using 4% paraformaldehyde for observation. Lung sections were counterstained with hematoxylin and eosin and imaged under a microscope. The primary tumors and parts of the lung tissue were surgically removed and fixed for F4/80 and CD206 IHC staining. All procedures were conducted in accordance with the China Experimental Animal Ethics Committee. Images were obtained with a microscope (objective: ×20; NA: 0.40; distance: 1.2 mm).

### ELISA Assays

The total amount of IL-4 was measured using a commercially available ELISA kit (Dakewe, Shenzhen, China) according to the manufacturer’s instructions. The samples were analyzed in triplicate. Absorbance was measured at 450 nm using a microplate reader. Standard curves were generated using the standards. The absorbance of wells containing only medium was used as the blank.

### Statistical Analysis

Data were expressed as mean ± standard error of the mean. Statistical analyses were performed with SPSS version 23.0. Statistical significance was assessed by ANOVA followed by the Tukey’s test with *p* < 0.05 considered statistically significant. Survival curves were analyzed using the Kaplan-Meier method.

## Results

### ICJ Inhibited Breast Cancer Metastasis in the Mouse Model of Pulmonary Metastasis

**Figure 1:**
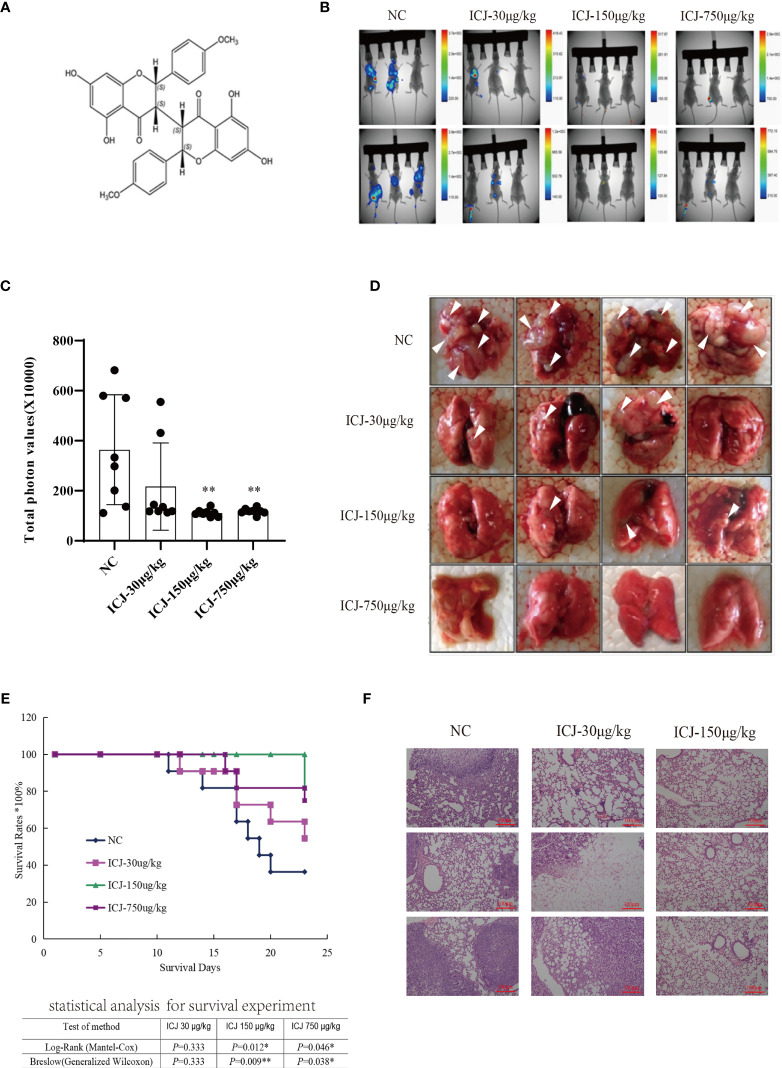
ICJ inhibits breast cancer metastasis *in vivo*. **(A)** The chemical structure of Chamaejasmenin B (ICJ). **(B)** Detection of metastasis through small animal imaging system. The images shown in the figure were obtained from 8 representative mice and taken on the 20th day. **(C)** Analysis of the total photon values. **(D)** General observation of lung metastasis. In the model group, images show a large area metastatic tumor in the negative control group. **(E)** Analysis of the survival period. **(F)** Hematoxylin and eosin staining (H&E) were used to analyze the intensity of metastasis. *p < 0.05 vs. NC, **p < 0.01 vs. NC, *N* = 8.

In a previous study (22, 23), we isolated and identified the structure of ICJ (**Figure 1A**) from *Stellera chamaejasme* L. In this study, we firstly evaluated the effects of ICJ on breast cancer metastatic outgrowth *in vivo* by directly injecting 4T1 cells into the tail vein of female Balb/c mice. As shown in **Figures 1B, C**, in the negative control group, the metastatic imaging signal was stronger in the lung tissue compared with in the bone, brain, liver, and other metastatic target organs. In comparison, ICJ treatment partially suppressed the outgrowth of lung metastases, as represented by the reduced total photon values in the ICJ-treated groups. Morphological observation (**Figure 1D**) indicated that detectable metastatic clones were widely spread in the lungs, leading to consolidation in most of the lungs in the modeling group. The survival period (**Figure 1E**) was evaluated on the 25th day after modeling. Based on HE staining (**Figure 1F**), the lung tissues were severely damaged by malignant metastatic outgrowth in the absence of ICJ, and ICJ provided significant protection.

In summary, based on the pulmonary metastasis mouse model, ICJ can significantly block metastatic outgrowth in highly invasive breast cancer.

### ICJ Influenced Macrophage Polarization and Infiltration *In Vivo*

**Figure 2:**
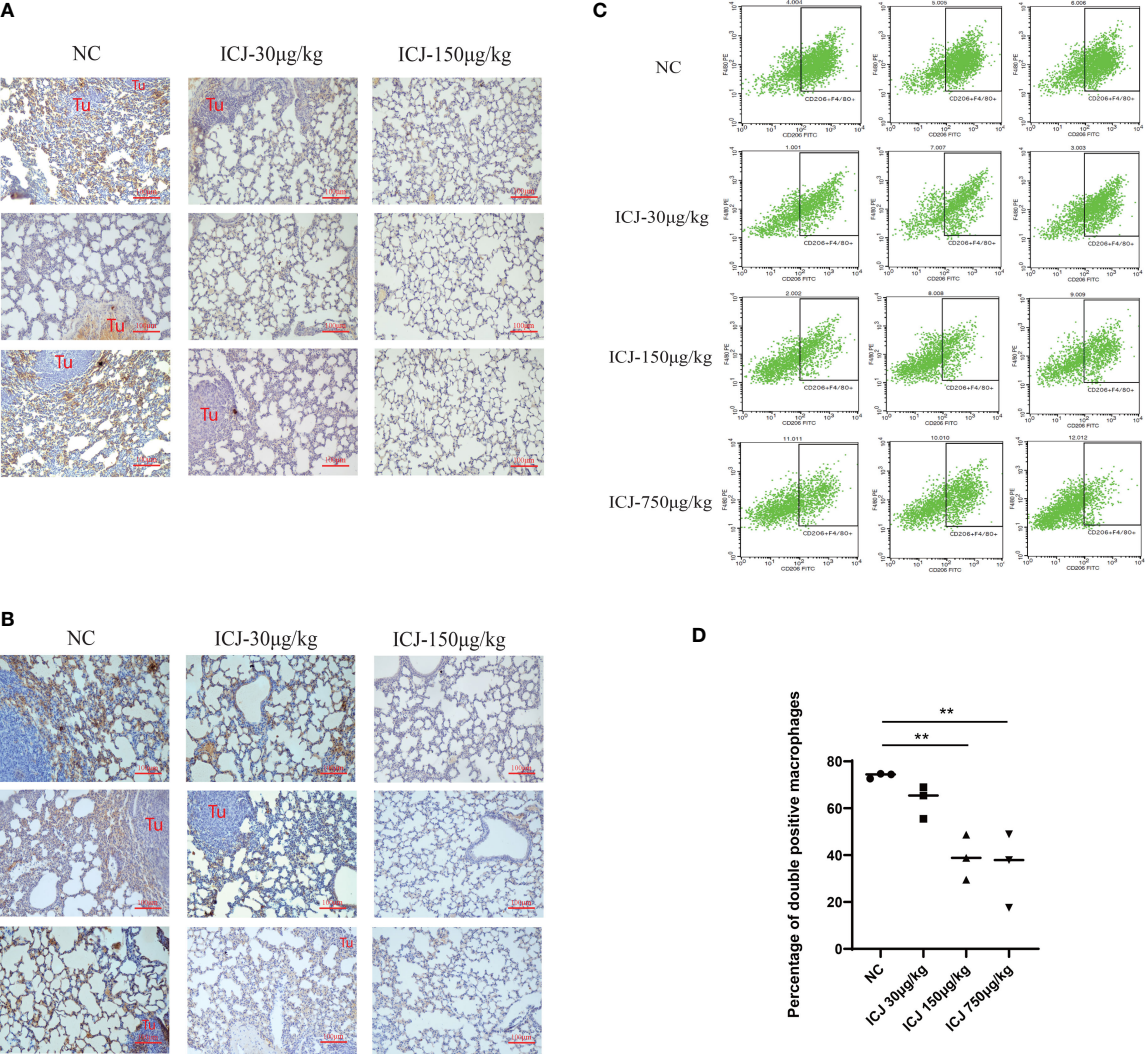
ICJ influenced macrophage polarization and infiltration *in vivo*. **(A)** The level of F4/80^+^ detected by IHC for evaluating macrophage infiltration in metastatic samples. **(B)** The level of CD206^+^ detected by IHC for evaluating macrophage polarization in metastatic samples. **(C, D)** The quantification of F4/80^+^CD206^+^ cells from metastatic samples was demonstrated with or without ICJ by flow cytometry. ICJ efficiently reverses the M2 macrophage polarization in metastatic sites. **p < 0.01 vs. NC, *N* = 3.

Leading by the close correlation between TAM and metastasis, the functional outcomes of tumor–macrophage interactions in the presence of ICJ was then evaluated *in vivo*. Firstly, the status of macrophages from metastatic samples was examined by IHC. **Figure 2A** shows that ICJ can control TAM infiltration at the metastatic site. Meanwhile, the amount of M2 macrophages that infiltrated the metastatic tissue was largely decreased by ICJ (**Figure 2B**), implying that the distribution of M2 macrophages, which were preferentially located near the tumor site, was altered by ICJ polarization of M2 macrophage, which were the dominant phenotype of macrophage in malignant microenvironment, was reversed by ICJ. We then further examined the F4/80 and CD206 expression in double-positive M2 cells of the metastatic samples by FACS analysis. Remarkably, this showed that TAMs are prone to the M2 phenotype, and that ICJ can control M1/M2 ratio at the metastatic site (**Figures 2C, D**).

### ICJ Directed M1 Macrophage Polarization Under Tumor-Associated Conditions *In Vitro*

**Figure 3:**
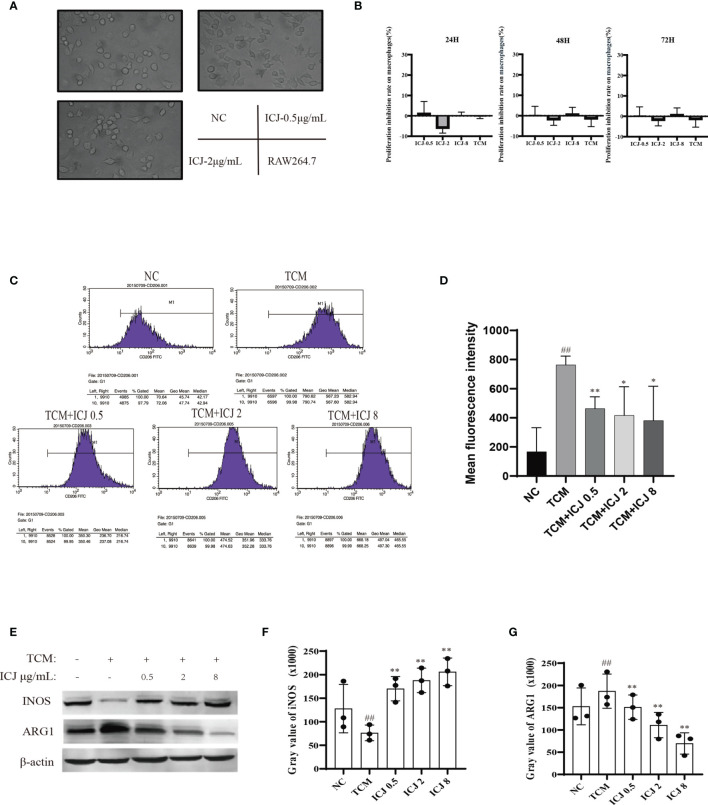
ICJ directed M1 macrophage polarization under tumor-associated conditions *in vitro*. **(A)** The morphological observation of RAW264.7 cells treated with ICJ. Images were collected in 24 h (objective, ×20; NA: 0.40; distance: 1.2 mm). **(B)** MTT assay for ICJ- and TCM-treated RAW 264.7 cells. Optical density values of 570 nm (OD570) were measured on the 24, 48, and 72 h, respectively. **(C)** The representative result of the inhibitory effect of ICJ in the regulation of M2 polarization marker CD206 in TCM- and ICJ-treated RAW264.7 cells by flow cytometry analysis. **(D)** The statistical analysis of mean fluorescence intensity of CD206 from 4 independent repeats in RAW264.7. **(E)** Western blot analysis of INOS and ARG1 in ICJ-treated RAW 264.7 cells for 24 h. **(F, G)** Statistical analysis of INOS and ARG1 gray values. ^##^p < 0.01 vs. NC, *p < 0.05 vs. TCM, **p < 0.01 vs. TCM, N = 3.

The effects of ICJ on macrophage polarization were further evaluated by morphological observation. As shown in **Figure 3A**, comparing with the small and rounded cell shape in negative control group, the cell size was dramatically increased with elongated pseudopodium in the presence of ICJ. Additionally, ICJ treatment led to an obvious increase in the adherent condition. This result suggest that macrophages may be a potential responsive cell for ICJ, which is worthy of further detailed analysis.

Based on the above morphological observations, we next determined the optimal doses of ICJ for *in vitro* experiments. Based on MTT assay (**Figure 3B**), ICJ concentrations lower than 10 μg/mL did not produce cytostatic interference. Therefore, 0.5–8 μg/mL ICJ was chosen as the nontoxic dosage range and used in further experiments.

As introduced above, the TME includes a complicated network of cellular interactions. Under this model, the surface expressions of molecular marker for M2 were detected by FACS in the presence of the tumor-conditioned medium (TCM). In line with previous studies, TCM significantly disturbed the polarization balance of macrophages by inducing the M2 phenotype. In contrast, the expression of CD206, a marker of M2 polarization, was reduced in the ICJ-treated group, indicating a shift away from M2 polarization (**Figures 3C, D**). This finding supports the morphological evidence, suggesting that ICJ can regulate macrophage polarization.

In keeping with the FACS data, Western blot analysis also suggested that the expressions of key markers of M1 or M2 were affected by ICJ treatment. As shown in **Figures 3E–G**, iNOS was inhibited by TCM, suggesting a compromised immune microenvironment. In contrast, the decrease of iNOS expression was reversed by ICJ in a dose-dependent manner. Accordingly, ICJ also reduced the expression of ARG1, a marker of M2 polarization.

### ICJ Reduced Clone Formation in TCM-Polarized M2 Macrophages and Inhibited PD-L1 Expression

**Figure 4:**
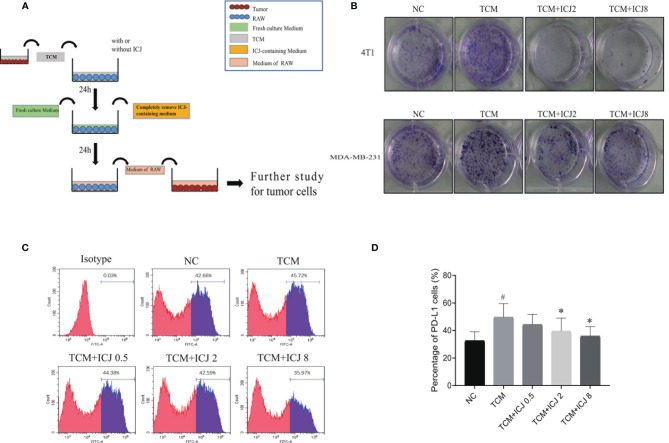
ICJ neutralized the enhanced clone formation effects of TCM-polarized M2 and inhibits PD-L1 expression. **(A)** The protocol for ICJ efficacy on tumor-macrophage interaction. **(B)** Colony formation assay in 4T1 and MDA-MB-231 cells. **(C, D)** The expression of PD-L1 in RAW264.7 cells treated with TCM and TCM-ICJ were examined by flow cytometry. ^#^ *p* < 0.05 vs. NC, ^*^ *p* < 0.05 vs. TCM, *N* = 3.

To further analyze the effects of ICJ on tumor cell, we established the conditioned medium transfer model (the experimental protocol is graphically depicted in **Figure 4A**). In detail, TCM with or without ICJ was firstly added into RAW 264.7. After 24 h, we totally removed the ICJ containing medium and added fresh culture medium into RAW 264.7 cell. After another 24 h, we used this secondary RAW 264.7-conditioned medium to culture tumor cell and the clonal proliferation potential of cancer cell was detected within each group. Based on above method, the pharmacological efficacy of ICJ from macrophage to tumor cell can be specifically detected, fully excluding the direct influence of ICJ on tumor cell by using post-ICJ-conditional medium.

Inspired by the ability of ICJ to combat TCM-induced M2 polarization, we attempted to clarify how ICJ affects tumor–macrophage interactions. Compared with the nontreated or TCM-treated groups, the tumor colony numbers were dramatically reduced by ICJ treatment in a dose-dependent manner (**Figure 4B**). Thus, in addition to rebalancing the M1/M2 ratio, pretreating macrophages with ICJ had a potent antiproliferative effect on highly invasive breast cancer cells. Therefore, we hypothesized that ICJ blocked the promalignant crosstalk between macrophages and tumor cells in the TME.

Programmed death ligand 1 (PD-L1) is a critical regulator of M2 polarization in macrophages. PD-L1 expression in macrophages drives the polarization of macrophages toward the M2 phenotype. We detected the surface expression of PD-L1 by flow cytometry. Compared with the TCM-treated group, ICJ treatment reduced PD-L1 expression (**Figures 4C, D**). Recent studies have shown that PD-L1 promotes cancer cell growth and proliferation *via* mTOR signaling (24). Meanwhile, blocking PD-L1 enhances cancer immunotherapy by regulating macrophage polarization (25, 26); this may be a potential antitumor mechanism of ICJ.

### ICJ Impairs IL-4-Induced M2 Polarization Through the AKT/mTOR Pathway

**Figure 5:**
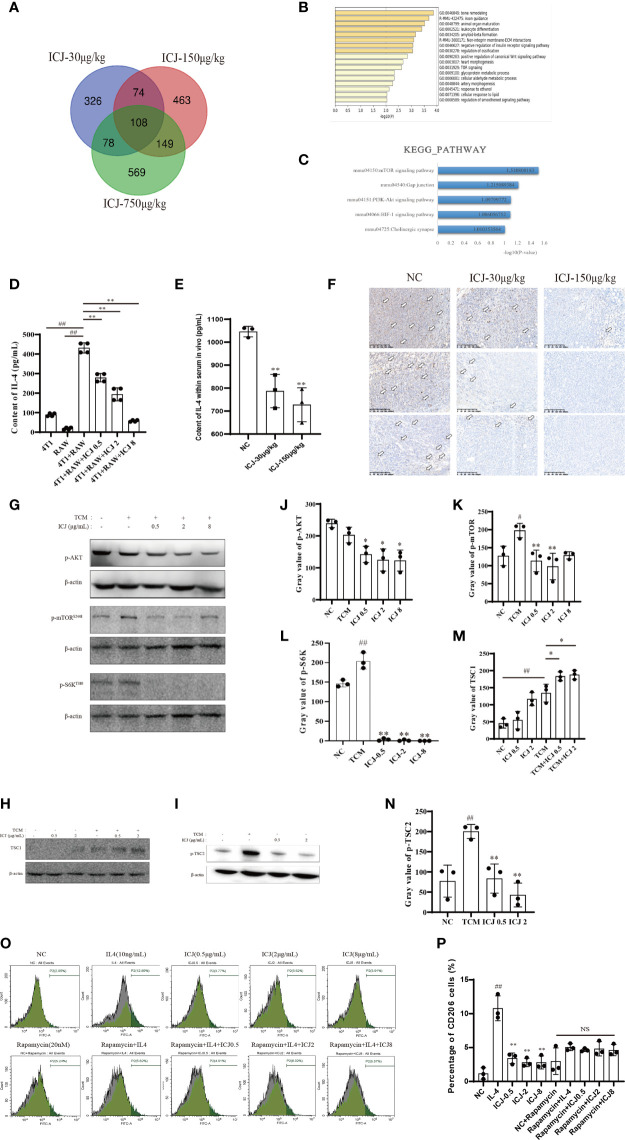
ICJ Impairs IL-4-induced M2 polarization through the AKT/mTOR pathway. **(A)** The Venn diagram captured 108 differentially expressed intersection genes in the ICJ-30 μg/kg, ICJ-150 μg/kg, and ICJ-750 μg/kg groups. **(B**, **C)** GO and KEGG pathway analyses on the 108 differential genes. The results indicated that the differential genes were significantly enriched in PI3K-Akt signaling pathway. **(D)** IL-4 expression of RAW 264.7 cells in conditional medium transfer model was inhibited by ICJ significantly. **(E)** The changes of IL-4 within serum *in vivo*. **(F)** The level of IL-4 detected by IHC of tumor. **(G)** Detection of p-AKT, p-mTOR, and p-S6K expression in ICJ-treated RAW 264.7 cells for 24 h. **(H**, **I)** Detection of TSC1 and p-TSC2 expression in ICJ-treated RAW 264.7 cells for 24 h. **(J**–**N)** Statistical analysis of p-AKT, p-mTOR, p-S6K, TSC1, and p-TSC2 gray values, respectively. **(O**, **P)** M2 marker CD206 in IL-4, IL-4+ICJ, and IL-4+ICJ+rapamycin-treated cells were examined by flow cytometry. NS, No Significant; ^#^ *p* < 0.05 vs. NC, ^##^ *p* < 0.01 vs. NC, ^*^ *p* < 0.05 vs. TCM, ^**^ *p* < 0.01 vs. TCM, *N* = 3.

To reveal the molecular mechanism by which ICJ hinders the metastatic outgrowth of breast cancer, the molecular network involved was explored through genome-wide microarray screening followed by bioinformatics-based experimental verification. We first obtained and analyzed the expression profiles of mRNAs from three groups of tumor tissues by microarray screening. We then identified 108 intersect genes that were differentially expressed in these three groups based on a Venn diagram (**Figure 5A**). Next, we performed gene ontology (GO) and KEGG pathway enrichment analyses using DAVID and KOBAS 3.0 to understand the potential functions of the 108 dysregulated mRNAs involved in tumorigenesis. Our analysis revealed the significantly enriched GO terms and enriched KEGG pathways of the mRNAs. mRNAs regulated by ICJ were significantly enriched in the signaling related to cell proliferation, growth, and motility (**Figures 5B, C**). Among them, mTOR was implied as the most-responsive pathway for ICJ.

Based on the above results, we hypothesized that the regulation of the PI3K-AKT-mTOR pathway might be the key mechanism in response to ICJ treatment. Within this pathway, IL-4 is a master regulator that participates in the polarization of macrophages to the M2 phenotype. Firstly, we examined the expression of IL-4 to determine the mechanism underlying the reduced M2 polarization in ICJ-treated macrophages. As confirmed by ELISA (**Figure 5D**), treatment with ICJ inhibited the expression of IL-4 under tumor-associated conditions. This result was consistent with the changes of IL-4 within serum and IHC *in vivo* (**Figures 5E, F**).

Next, we evaluated the phosphorylation level of AKT, which is the downstream kinase of IL-4/IL-4R. We detected ICJ inhibited the TCM-induced activation of AKT (**Figures 5G, J**). Additionally, mTOR phosphorylation and S6k, another key molecule downstream of mTOR, were evaluated by Western blot. As expected, ICJ significantly inhibited pho-mTOR and pho-S6k (**Figures 5G, K, L**). Noteworthy, ICJ increased the expression of TSC1 (**Figures 5H, M**), a natural inhibitor of the mTOR pathway and decreased the phosphorylation level of TSC2 (**Figures 5I, N**), molecularly suggested that, in the presence of ICJ, the mTOR inhibition can be achieved by the enhanced function of TSC complex.

To prove that macrophage polarization induced by ICJ was mTOR dependent, mTOR was inactivated by using the chemical inhibitor rapamycin. Under mTOR-blocked conditions, ICJ failed to reverse M2 macrophage polarization (**Figures 5O, P**). In conclusion, our data supported our hypothesis that ICJ inhibited M2 polarization in malignant microenvironment through AKT pathway, which highly relied on mTOR.

## Discussion

Metastasis is the leading cause of death for most cancer patients. Immunologically, the surveillance and clearance of malignant cells, which are conducted primarily by immune cells that infiltrate into tumor tissues, are severely disturbed, contributing to the progression of metastasis. Within such tumor-friendly “soil,” TAMs have numerous protumoral functions that support the colonization and metastasis of human breast cancer cells (27). Based on the current study (28–30), targeting macrophage polarization could be an effective therapeutic strategy for antimetastasis therapy.

Although the depletion of macrophages shows any beneficial effects in the inhibition of tumor growth and metastasis (31), the nonselective depletion of macrophages may cause many unexpected immune-suppressive effects, as macrophages also have unique abilities for pathogen clearance or injury repair (32). Recent studies hold the view that the imbalanced M1/M2 polarization, rather than the increased capacity of macrophage pool, forms the basis of immune disturbance and as a result, supports metastasis (33). Therefore, the screening and identification of candidate that can rebalance the pathological polarization in TAMs is a promising strategy to prevent metastasis. SCL is an effective herbal medicine that is widely applied in treating various malignancies. In previous studies, we revealed the antimetastatic efficacy of SCL extract (22, 23). In the present study, we further proved the molecular mechanism through which SCL extract combat metastasis. In addition, we clarified the pharmacological effects of ICJ on the TME.

Based on the “growing while metastasizing” model, metastasis in breast cancer is initiated in the early phase of the disease, even earlier than the primary tumors can be clinically detected. Tumor cells, which have been metastasized in foreign tissues, can remain dormant for years. These metastasized but dormant tumor cells are highly resistant to most currently established chemotherapeutics, highlighting the importance of early, outgrowth-specific interventions, which require additional development. Thus, it is critical to investigate potential therapeutic compounds with a wider treatment time window and higher specificity against metastatic colonizing tumor cells. Our results demonstrate that ICJ has the potential to block outgrowth during metastasis by reversing imbalanced macrophage polarization. This suggests that ICJ treatment has taken the micrometastasis survived in the foreign tissues into the scope of intervention and thereby, expanded the time window for the restriction of metastasis happened in the early phase.

IL-4 is an important cytokine that activates the AKT/mTOR signaling pathway. The differentiation of infiltrated macrophages into the TAM phenotype *via* IL-4 cytokines is well known. Therefore, we hypothesized that the IL-4 signaling pathway could be a therapeutic target for inhibiting TAM-induced pro-metastatic colonization. As for the discrepancy of p-mTOR and other results, we postulated that the molecular network regulating macrophage polarization is highly complicated, existing crosstalk among multiple pathways. Considering this, instead of the influence on mTOR alone, the higher concentration of ICJ might induce the integrated disturbance for the whole polarization network (e.g., JAK2-STAT6) or even initiate the negative feedback mechanism for mTOR. The scientific explanation needs further detailed analysis. The scientific explanation needs further detailed analysis. As mentioned above, STAT6 is the master regulator of M2 activation in response to IL-4. Blocking the JAk2-STAT6 pathway may be a possible mechanism by which the ICJ inhibits M2 differentiation *in vitro*. In a future study, we could test this idea by investigating the effect of ICJ on STAT6 genetically deficient mice and its association to macrophage polarization in tumor burden mice.

In addition, in our future study, by using macrophage-deficient model and M1 polarization energy model, we firstly plan to reveal the dependency and interaction of ICJ to macrophage polarization. Of note, the regulatory relationship between macrophage polarization and angiogenesis, which has been proved by previous study, should be performed to obtain more information about the responsive network to ICJ treatment.

In summary, for the first time, we demonstrated that ICJ extracted from the traditional Chinese herb *Stellera chamaejasme* L. reduced metastatic colonization in breast cancer cells by inhibiting the AKT/mTOR pathway in TAMs. Additionally, we confirmed that ICJ inhibited the expression of IL-4 and reversed M2 macrophage polarization in a tumor-associated condition model *in vitro*. Treatment with ICJ not only attenuated tumor colonization, it also inhibited early metastasis in a 4T1 mammary carcinoma mouse model. This study highlights the importance of the IL-4-activated AKT/mTOR signaling pathway in TAM differentiation and suggests that the inhibition of this pathway may be an effective way to block TAM-induced prometastatic colonization.

## Data Availability Statement

The original contributions presented in the study are included in the article/supplementary material. Further inquiries can be directed to the corresponding authors.

## Ethics Statement

The design of animal study was guided by the policy from China Experimental Animal Ethics Committee. All the related experiments were reviewed and approved by the Laboratory Animal Ethics Committee of the Institute of Chinese Materia Medica.

## Author Contributions

XZ provided the theoretical hypothesis of this study. QL and LS performed most part of the experiments. LL contributed significantly to analysis and manuscript preparation. LS and QL performed the data analysis and wrote the manuscript. Other authors revised the data and provided constructive suggestions. All authors contributed to the article and approved the submitted version.

## Funding

The present study was supported by the China Academy of Chinese Medical Sciences Innovation Fund (grant numbers: CI2021A05105); Special training program for outstanding young scientific and technological talents of China Academy of Chinese Medical Science (grant numbers: ZZ13-YQ-044); The National Science and Technology Major Projects for Major New Drugs Innovation and Development (grant numbers: 2017ZX09101002-002-002); “the Belt and Road” Cooperation Project of China Academy of Traditional Chinese Medicine (grant numbers: GH201914); The National Science Foundation for Young Scientists of China (grant numbers: 81303272).

## Conflict of Interest

The authors declare that the research was conducted in the absence of any commercial or financial relationships that could be construed as a potential conflict of interest.

## Publisher’s Note

All claims expressed in this article are solely those of the authors and do not necessarily represent those of their affiliated organizations, or those of the publisher, the editors and the reviewers. Any product that may be evaluated in this article, or claim that may be made by its manufacturer, is not guaranteed or endorsed by the publisher.
